# Assessing the Role of Gaze Tracking in Optimizing Humans-In-The-Loop Telerobotic Operation Using Multimodal Feedback

**DOI:** 10.3389/frobt.2021.578596

**Published:** 2021-10-04

**Authors:** Joseph Bolarinwa, Iveta Eimontaite, Tom Mitchell, Sanja Dogramadzi, Praminda Caleb-Solly

**Affiliations:** ^1^ Bristol Robotics Laboratory, University of the West of England (UWE), Bristol, United Kingdom; ^2^ Creative Technologies Lab, University of the West of England (UWE), Bristol, United Kingdom

**Keywords:** peripheral vision, gaze, fixation, haptics, feedback modalities, collaboration, telerobotic assistance, teleoperation

## Abstract

A key challenge in achieving effective robot teleoperation is minimizing teleoperators’ cognitive workload and fatigue. We set out to investigate the extent to which gaze tracking data can reveal how teleoperators interact with a system. In this study, we present an analysis of gaze tracking, captured as participants completed a multi-stage task: grasping and emptying the contents of a jar into a container. The task was repeated with different combinations of visual, haptic, and verbal feedback. Our aim was to determine if teleoperation workload can be inferred by combining the gaze duration, fixation count, task completion time, and complexity of robot motion (measured as the sum of robot joint steps) at different stages of the task. Visual information of the robot workspace was captured using four cameras, positioned to capture the robot workspace from different angles. These camera views (aerial, right, eye-level, and left) were displayed through four quadrants (top-left, top-right, bottom-left, and bottom-right quadrants) of participants’ video feedback computer screen, respectively. We found that the gaze duration and the fixation count were highly dependent on the stage of the task and the feedback scenario utilized. The results revealed that combining feedback modalities reduced the cognitive workload (inferred by investigating the correlation between gaze duration, fixation count, task completion time, success or failure of task completion, and robot gripper trajectories), particularly in the task stages that require more precision. There was a significant positive correlation between gaze duration and complexity of robot joint movements. Participants’ gaze outside the areas of interest (distractions) was not influenced by feedback scenarios. A learning effect was observed in the use of the controller for all participants as they repeated the task with different feedback combination scenarios. To design a system for teleoperation, applicable in healthcare, we found that the analysis of teleoperators’ gaze can help understand how teleoperators interact with the system, hence making it possible to develop the system from the teleoperators’ stand point.

## 1 Introduction

There are still several challenges for effective telerobotic operation, particularly in tasks that require a high degree of teleoperator agility for manipulation. Teleoperators are required to process information relating to the status of the robot and remote environment which can result in high cognitive load and fatigue. Different application domains bring specific requirements which can increase the teleoperator’s cognitive workload. For example, in our target application domain, providing remote assistance in a social care context, teleoperators are required to interact socially with the remote service user in addition to carrying out the task.

This study reports on the use of gaze data to explore how participants interacted with a teleoperation system and, in particular, how gaze data (gaze duration and fixation count) can be used to understand the effects of different feedback modalities. Since we are interested in the use of teleoperated robots in a social care context, we examine the effect of social interaction between the teleoperator and service user. In the research work reported in this study, video feedback in combination with verbal, haptic, and/or peripheral visual feedback were provided in different feedback combination scenarios to improve task performance and reduce the workload. The hypotheses made before the study was conducted are listed below:

**H1:** Gaze duration and fixation count toward control instructions will significantly decrease from Attempt 1 to Attempt 7 showing learning effects.

**H2:** Gaze duration and fixation count can be used to measure the impact of feedback modalities on teleoperator performance (task completion time and success or failure of the task).

**H3:** Gaze duration and fixation count can be used to determine teleoperator distraction caused by different feedback scenarios.

**FIGURE 1:**
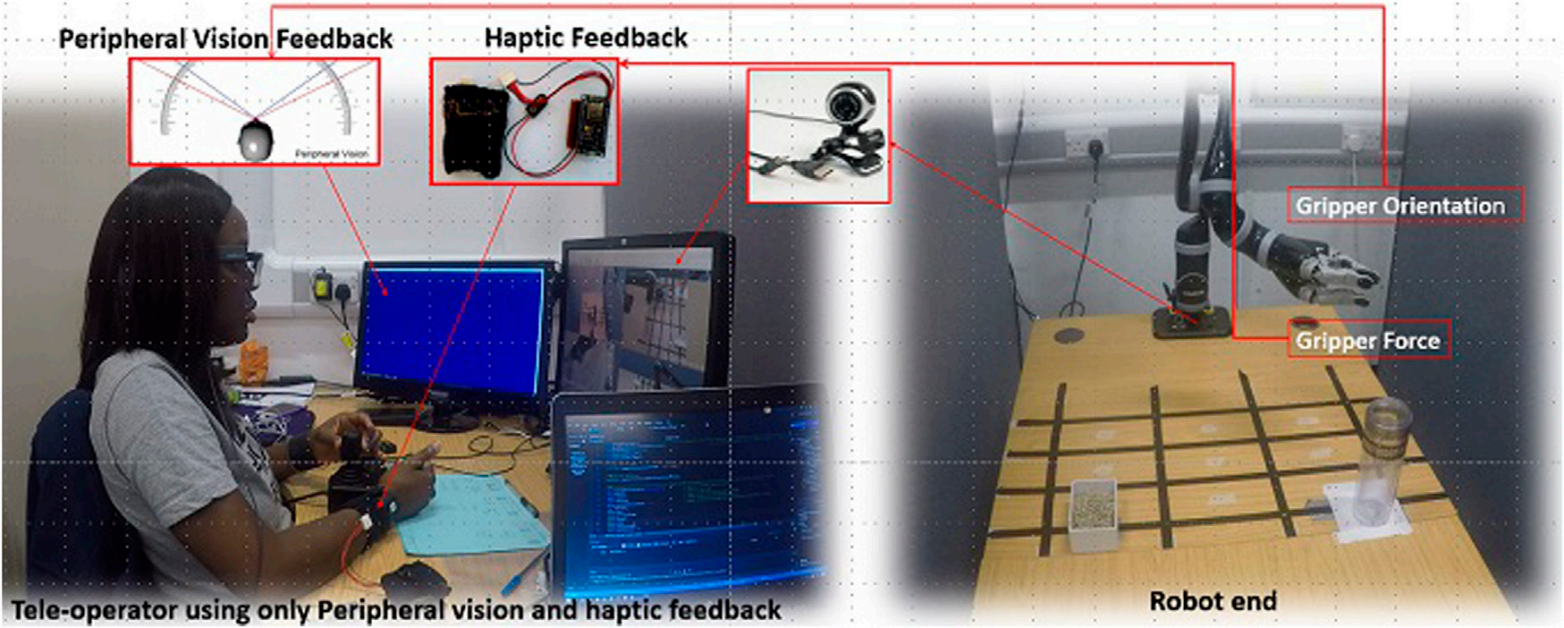
Experimental setup.

The study presented here extends the report presented by Bolarinwa et al. (2019) focusing specifically on the use of gaze tracking to assess how participants employ a combination of visual, haptic, and verbal feedback modalities to assist them during different stages of an object manipulation task, while also interacting with a remote service user (Figure 1).

The gaze tracking data was captured as participants completed a multi-stage task: grasping and emptying the contents of a jar into a container. Our aim was to determine if workload can be inferred by combining gaze duration, the fixation count, task completion time, and the complexity of robot motion (measured as the sum of robot joint steps) and comparing these during the different stages of the task.

## 2 Related Work

Teleoperated robots have also made it possible to carry out tasks in extreme environments that, as a result of logistical or financial constraints, are otherwise inaccessible (Tsitsimpelis et al., 2019). The introduction of embodiment within the robot, as well as immersion in the remote environment, improved teleoperation experience and success (Pamungkas and Ward, 2015). This provides an equivalent experience of an actual environment (Hernandez et al., 2017). An efficient user interface with dynamic multiple sensory feedback and actuator control is thus important to facilitate multimodal perception using the human innate sensory abilities. The need for feedback, as well as the type of feedback, however, depends on the context in which the teleoperated robot is applied. This study focuses on assistive robots.

### Assistive Robots

One of the functions of assistive robots is helping the elderly and people living with disabilities with their activities in daily life (Begum et al., 2013) (Góngora Alonso et al., 2019). Assistive robots that are designed to perform more diverse tasks also encourage greater collaboration with humans, particularly in terms of social interaction which helps to build trust and empathy (Langer et al., 2019) (Lv et al., 2020) (Begum et al., 2015). The need for social interaction and the unpredictable nature of living environments makes a case for the introduction of teleoperated assistive robots (Bolarinwa et al., 2019). The safety of service users is very important for human-robot interaction, especially for uncontrolled environments which may make teleoperation more difficult. As a result, a wide range of feedback modalities are employed to convey complex information about the status of the robot and the remote environment to teleoperators.

### Multimodal Feedback

Multimodal feedback in teleoperation involves the use of more than one form of feedback during a teleoperation task. Vitense et al. (2002) investigated how unimodal, bimodal, and trimodal feedback affect the complex direct manipulation performance of fully sighted users. Investigations were carried out on auditory feedback, haptic feedback, and visual feedback. During the task, workload was measured objectively and subjectively, and results showed that multimodal feedback improves the performance of participants that are fully sighted. Results also showed great potential for participants with visual impairments. Luz et al. (2018) employed the use of a haptic interface to display the traction states of a teleoperated unmanned ground vehicle (UGV) to human operator through different types of tactile stimuli provided by three haptic devices (E-Vita, traction cylinder, and vibrotactile glove). A teleoperation interface was used to display images from the on-board camera to participants and a 3D Connexion Space Navigator 6-DOF joystick was used to control the UGC. They evaluated the extent to which the feedback modality improves the user situation awareness in regard to the traction state of the UGV. Loss of traction was measured using a laser-based traction detection module. Traction states were displayed to the human operators *via* the haptic devices. Improvements were found in the comprehension of the UGV’s traction state when a combination of the vibrotactile glove and the traction cylinder were used.

### Cognitive Overload

The use of multiple feedback modalities can improve teleoperation performance but may also result in an increased cognitive load (Triantafyllidis et al., 2020). Although the provision of additional feedback of the robot’s status and its work environment increases situational awareness (hence making it easier to carry out tasks), the increase in the amount of feedback information that the teleoperator has to process may increase the teleoperator’s cognitive load. Applying the multiple resource theory (Wickens, 2002) in the context of teleoperated assistive care, teleoperators will carry out dual tasks by engaging in social interaction with the service users while also carrying out tasks. For tasks where the teleoperator might be providing remote assistance to a person, we need to consider even more carefully the cognitive load that specific type of feedback might have on a teleoperator, as the teleoperator might also have to engage socially with the remote person they are assisting to build trust and empathy (Langer et al., 2019). Triantafyllidis et al. (2020) assessed how multimodal interfaces (audio, haptic, and visual feedback) can improve teleoperation by subjectively measuring the cognitive workload across the different feedback modalities. Visual feedback incorporating monocular vision with the display monitor accounted for significantly higher perceived workload than stereoscopic vision seen through the virtual reality head-mounted displays. In an attempt to provide detailed frame of view for precise teleoperation, Mizukoshi et al. (2020) investigated the use of an automatic zoom method to improve teleoperation performance. This method ensures that images were zoomed when a robot arm entered a work area, but as a result of lack of consideration for the intentions of teleoperators as well as zoom levels, resultant effects of high cognitive workload and motion sickness occurred (Mizukoshi et al., 2020). The study carried out by Wang et al. (2018) highlights the importance of considering human-centric metrics in the design of teleoperation strategies. They opined that while additional haptic feedback in the Cartesian space resulted in improved performance of a robotic needle steering, it may increase cognitive workload and cause muscle fatigue. Monitoring cognitive overload is therefore important owing to the effect it has on teleoperator performance. To predict and mitigate workload for training simulation, Jones et al. (2015) employed cognitive workload analysis to identify factors that cause cognitive overloading. Due to the fact that existing approaches allow for subjective assessment and may be susceptible to user physiology and environmental influences; Kosch et al. (2018) explored the use of gaze data for monitoring cognitive workload. They found that there were higher deviations of gaze points at higher cognitive workload levels during smooth pursuit eye movements for specific trajectory types (Jones et al., 2015). Jones et al. (2015) were able to predict cognitive workload through smooth pursuit with an accuracy of 99.5% between low and high workload using an SVM classifier. Using gaze data (gaze duration and the fixation count), we therefore set out to monitor cognitive workload for different stages of a teleoperation task in this study.

### Gaze Tracking

There are increasing numbers of applications that use gaze tracking, such as video games, as well as applications involving virtual reality (VR). For communication and interaction, humans exhibit non-verbal cues to signal intent and at the same time interpret behavioral cues of others to predict their intentions. In order to establish perceptual common grounds, people often use gaze cues to draw other people’s attention (Loth and De Ruiter, 2016). The cues are typically included as part of head movements, facial expressions, gestures, and eye gaze. This is especially important in collaborative scenarios, enabling humans to predict each other’s intentions and adjust their own actions accordingly (Huang et al., 2015). Therefore, understanding these natural cues may help facilitate better human-robot interactions when robots are introduced into the loop.

Gaze patterns have been used to predict human intention in various applications to date, either replacing or accompanying verbal expressions. In teleoperation, studies are being carried out to achieve monitoring and control using the operators’ eye gaze. A common problem that arises in eye tracking is the “Midas touch” (Jacob, 1990). This arises as a result of the difficulty in picking out commands from a continuous stream of inputs. One of the proposed solutions is the introduction of “dwell time” and additional input devices as triggering mechanisms. Dwell time constituted keeping the gaze within a particular area to substitute for the act of clicking (Latif et al., 2009). Eye tracking measures such as total duration of “dwell time”, number of dwells, number of options attended to, mean dwells on each option, and total dwell time on each option have been introduced to analyze gaze data and understand related cognitive and decision-making processes Gidlöf et al. (2013).

Teleoperation using gaze tracking can be accompanied by another mean of body tracking to achieve certain teleoperation modes. Chen et al. (2019) proposed a teleoperation method by combining gaze tracking and movements to teleoperate a KUKA youBot. By combining instantaneous states of the tracked gaze and hand motion, they were able to adapt the speed of the robot using eye-hand coordination. For applications that require minimal manual input, or in cases where users have motor disabilities, an eye gaze-tracking system to teleoperate a mobile robot (Carreto et al., 2018) proved to be a successful alternative to keyboard and mouse. However, a review of the system usability scale scores showed that more development on this kind of interface is needed (Carreto et al., 2018).

This study reports on utilizing gaze data to analyze participants’ interaction with a teleoperation system during a simple task. The teleoperation system employs a range of different feedback modalities (scenarios) including verbal collaboration, haptic feedback, video feedback, and peripheral vision to support teleoperation performance. With the provided video feedback, we explored the effect of different feedback combinations on teleoperator’s gaze. This further helped us to analyze how each participant used the available feedback which may also be used to infer the workload at different stages of the task. Gaze data gathered is defined by the areas of interest on the different camera views displayed to teleoperators as they carry out the task.

For all the scenarios employed in the study, video feedback was always provided to the participants.

## 3 Materials and Methods

This study builds on the research work presented in Bolarinwa et al. (2019), and the key elements of the method have been included here for clarity. Ethics approval for the study was obtained from the ethics committee of the University of the West of England, Bristol (UWE REC REF NO: FET.17.12.015).

### 3.1 Design

The study used a repeated measures design with seven scenarios (scenarios: S1 (video feedback only), S2 (video feedback and verbal collaboration), S3 (video feedback and peripheral vision), S4 (video feedback, peripheral vision, and verbal collaboration), S5 (video feedback and haptic feedback), S6 (video feedback, haptic feedback, and verbal collaboration), and S7 (video feedback, haptic feedback, peripheral vision, and verbal collaboration)). The scenarios were presented in the counterbalanced order between participants. As the study’s main aim was to investigate the role of gaze data in understanding how teleoperators interacted with the system, the independent variables were scenario, camera view, and the attempt number. The dependent variables were eye tracker data (gaze duration and the fixation count), and the results were controlled for participants’ gaming experience. Since the robot was controlled using a joystick, participants’ prior gaming and robot control experience was considered as an important factor that could give them advantage (lower task completion time or smoothness of gripper control) over those without prior gaming experience. Gaming experience was introduced to understand if participants with prior gaming experience would interact with the system differently relative to participants without prior gaming experience.

### 3.2 Study Setup

The study was conducted in the Assisted Living Studio in the Bristol Robotics Laboratory, at the University of the West of England, Bristol, United Kingdom. The study setup is shown in Figure 1. A 6-degrees-of-freedom JACO2 robot arm from Kinova (2019) was used in the study to carry out an assistive task that might be performed for a service user with mobility issues. Participants (teleoperators) controlled the arm using the robot’s joystick controller. Communication between the robot and the joystick is *via* a one Mbps CAN bus. Two levels of control system frequency are available: a high level (100 Hz) and a low level (up to 500 Hz) *via* APIs. The gripper orientation and robot arm joint movements were polled every 10 milliseconds. Figure 1 shows a participant controlling the JACO2 robot with a joystick. Camera views of the remote robot end were displayed on the screen (using 4 quadrants) directly in front of the participants. The chosen camera views show the same robot workspace views participants would have if each participant were to be present locally in the same physical location as the robot. The adjustments of the camera positions and the decision regarding the number of cameras used were made after the completion of a pilot study. The camera view in each quadrant was assigned randomly and kept constant for all participants. The gripper orientation polled from the robot was displayed to participants *via* the wrist worn haptic devices and the display screen in the participants’ left peripheral view. Tobii Pro Glasses 2 were worn by participants to track and record their gaze data as they carried out the task. The study protocol was clearly explained to participants before they repeatedly teleoperated the robot to pick up a jar filled with sunflower seeds, empty the contents of a jar into a container, and return the jar to its initial position. An instruction sheet that describes how the robot can be controlled with the joystick was provided for consultation by participants as they carried out the task. The experiment took 60 min to complete per participant. Scheduled breaks were taken by participants between each task repetition to eliminate the effects of fatigue on the performances of participants. Participants rested after each task, during which changes were made to the feedback scenarios and the system setup for the task. Additional rest time was given based on participants’ requests.

The role of the service-user was performed by the principal researcher. The principal researcher engaged with the participants in two ways. First, through social interaction, providing verbal feedback on how participants were performing during the task. Second, by signaling the end of each stage and whether the stage had been completed successfully or otherwise using a series of buttons designed for the study. The duration of each stage along with the total number of robot steps and instantaneous orientation of the robot gripper were logged by the software. The task was divided into four stages:

**Stage 1:** Free-space translation and rotation of the gripper from a defined position to a position and orientation close to the jar for successful grasp.

**Stage 2:** Grasping the jar and making free space translation to a position where its content can be emptied into another container.

**Stage 3:** Free space rotation and translation of the jar to empty its content into a container.

**Stage 4:** Free space translation and rotation of the emptied jar to its pick-up position.

**FIGURE 2:**
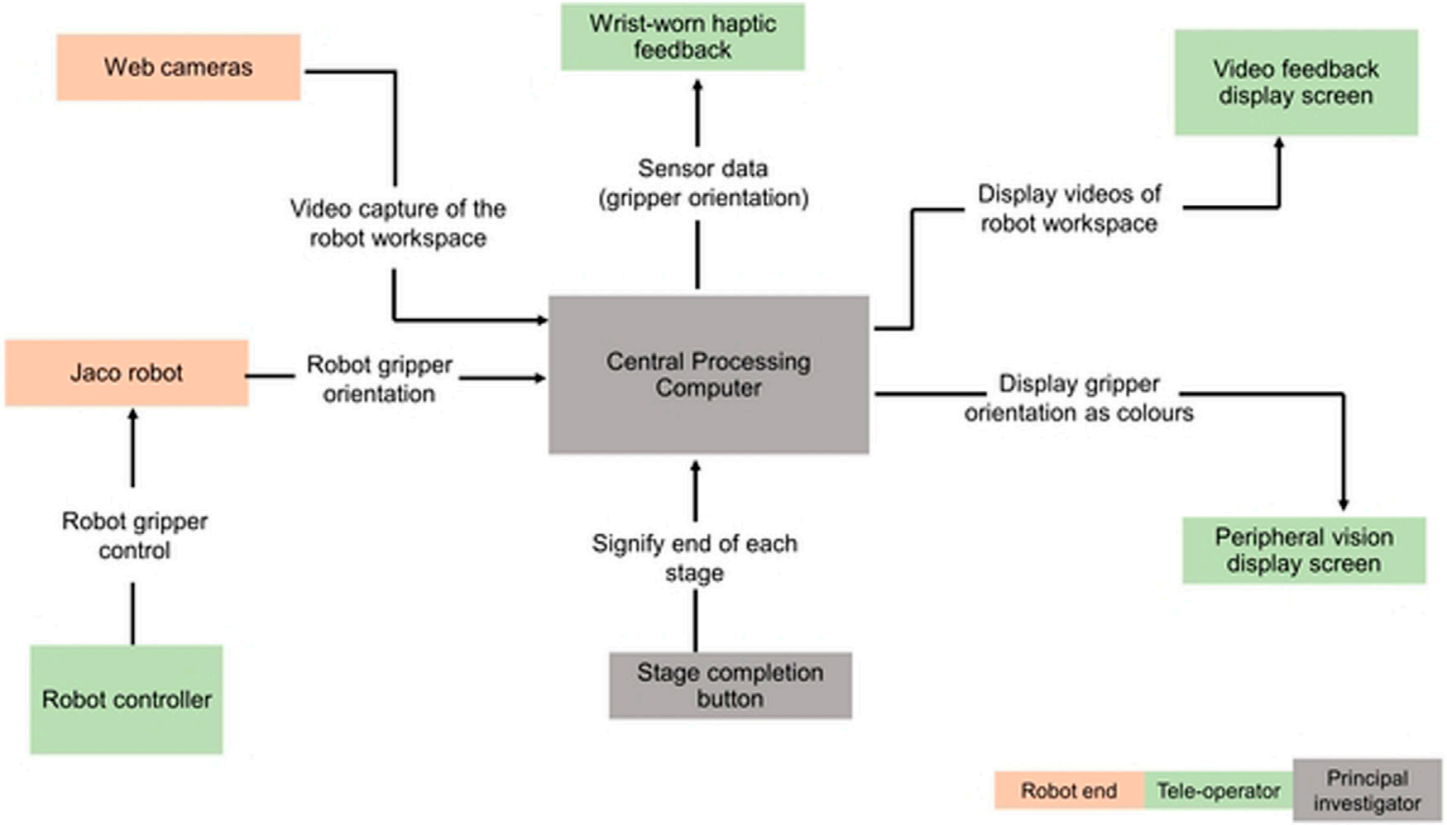
Setup block diagram.

The teleoperation task, comprising of all these stages, was repeated seven times by each of the participants with different feedback combination scenarios and verbal collaboration between participants (the teleoperator) and the principal researcher (service-user), as shown in Table 2. In Figure 2, each block shows the components of the study setup and the direction of information flow. The central processing block is a laptop computer which communicates with the JACO2 robot, polling the gripper orientation values from it and mapping the polled data to different feedback modalities.

In aiding a potential frail and vulnerable service-user, it is likely that the teleoperator and service-user will interact socially to make the experience pleasant and engaging. Interacting socially will require that the teleoperator communicates with the service-user and responds to non-verbal cues. As a result, feedback modalities were chosen that would not inhibit social interaction between the teleoperator and service user. The verbal feedback from the service user included providing additional information to the teleoperator that could help with the task and is also intended to make the service user feel engaged in the task completion. The feedback modalities employed are explained below.

### 3.3 Feedback Modalities Used in the Experiment

#### Video

**FIGURE 3:**
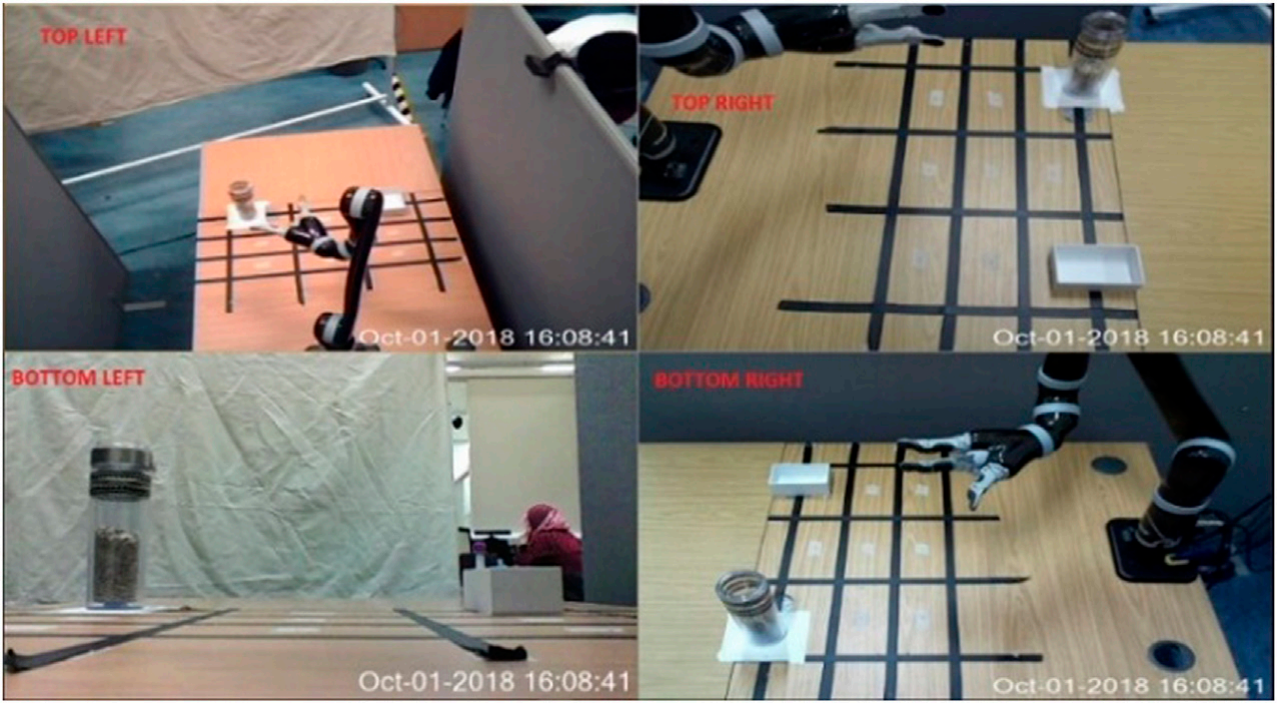
Screen shot of four different camera views.

Videos of the remote location were captured using four RGB web cameras and were displayed on four quadrants on a single screen, as shown in Figure 3. Video feedback was available continuously, featuring in every combination of feedback (scenarios). The top-left quadrant of Figure 3 shows the aerial view of the robot’s workspace, while the quadrant labeled “top-right” provides participants with the right view of the workspace. The “bottom-left” quadrant focusses on eye-level view of the workspace to make it possible to see the distance between the gripper and the table, as well as the placement of the jar on the table. The bottom-right quadrant shows the left view of the robot workspace.

#### Haptic

WiFi-enabled haptic wrist bands were used to provide information about the gripper orientation to participants. The wrist bands were custom-made for this study and incorporated 4 vibration motors located inside a soft sport wrist band. The amplitude of vibration of the motors increases as the gripper moves away from a set vertical position. In this study, haptic wrist bands were worn on each hand to signal left and right directions of movements of the gripper.

#### Peripheral Vision

Peripheral vision refers to the ability to see objects, movements, and changes in the environment outside of the direct line of vision (William, 2018). Information about the gripper orientation was presented as color changes on a second screen positioned in participants’ left peripheral field of view. Table 1 shows the gripper orientation values mapped to different orientations of the gripper and assigned colors for the peripheral vision screen.

**TABLE 1:** Robot gripper orientation scores.

Orientation position classification	Gripper orientation ranges (degrees)	Color representation	Assigned values
Aligned with the vertical axis of the jar	125–134, 482–492, −221–−231	Green	5
A little tilted to the left	134.5–140 (0.5–5° more than the upper-class boundary of the ‘aligned range’)	Light blue	2
Farther to the left from the aligned position	140.5–482 (0.5–41° more than the upper-class boundary of the ‘slightly tilted to the left ‘range)	Deep blue	0
A little tilted to the right	119.5–124.5 (0.5–5° less than the lower-class boundary of the ‘aligned’ ‘range)	Light red	2
Farther to the right from the vertical alignment	−221–120 (0.5–341° less than the lower-class boundary of the ‘aligned ‘range)	Deep red	0

#### Verbal Feedback

We introduced verbal collaboration to represent interactions between teleoperators and service users. In the study, the principal researcher (acting as the service user) provided verbal feedback on teleoperators’ performance as they carried out the task. This fits into a typical assistive care scenario where a career provides assistance using a teleoperated robot whilst socially engaging with the service user.

#### Feedback Combinations

Table 2 shows different combinations of feedback and collaboration with the “service user” that were used in the experiments. To ensure parity, the order of the feedback combinations was randomized using Latin Square counterbalancing. The combination of just peripheral feedback and haptic feedback was not included in the experiment because the overall time required for the experiment would have been impractical, given all the separate and paired combinations that were carried out; however, this has been planned for future experiments.

**TABLE 2:** Feedback scenarios.

Scenarios	Feedback and collaboration scenarios
1	Video feedback only
2	Video feedback and verbal collaboration
3	Video feedback and peripheral vision
4	Video feedback, peripheral vision, and verbal collaboration
5	Video feedback and Haptic feedback
6	Video feedback, haptic feedback, and verbal collaboration
7	Video feedback, haptic feedback, peripheral vision, and verbal collaboration

### 3.4 Participants

Invitations were sent out for voluntary participation across the university. After completing a consent form, 11 people participated in the study, five were male and six were female. Participants had a mean age of 29.5 (SD = 7.54), 2.36 mean years of robot experience (SD = 3.2), and mean years of gaming experience of 5.6 (SD = 7.63). Participants’ robot experience is a measure of participants’ familiarity with controlling or working (research or demonstration) with robots, measured in years. None of the participants reported color blindness, and of all participants, 10 participants were right-handed and only one participant was left-handed. Handedness was determined based on statements made by each participant. The setup was not affected in anyway by the left-handedness or right-handedness of the participants. Participants operated the robot controller with their dominant hands.

### 3.5 Analysis

We present analysis of participants’ gaze data recorded during the task. For all data recorded *via* the Tobii Pro Glasses, the percentage of fixation was 100. The Tobii Pro software was used to extract the gaze duration and the fixation count for defined regions of interest into an Excel spreadsheet. The regions of interest include a video feedback display screen (with four quadrants), robot control instruction sheet, robot controller joystick, and peripheral display screen. As eye gaze was continuous, data with normal distribution and parametric tests for inferential statistics were carried out using the IBM SPSS statistics software. The Shapiro–Wilk test for normality was carried out on the captured data. The data was normally distributed, and therefore, our small sample size (up to samples as small as 5) is appropriate for a parametric test (Winter 2013). Furthermore, with current sample size (N = 11), we compute the statistical power of 0.80 which is appropriate for generalization.

#### Recorded Parameters

The dependent variables measured in the study were the overall robot steps (calculated as the sum of the number of discrete robot arm joint movements in x, y, and z planes) taken to complete the task, gaze data, robot gripper orientation, time needed to complete the task, and subjective ratings of ease of use and usability of the system (system usability scale, Brooke (2004)). The robot arm joint movements and gripper orientation were polled in software from the robot using the robot’s application programming interface (API). The independent variables were feedback combinations S1–S7 (Table 2). During experiments, we recorded each stage’s completion, success, and the task completion time, the participants’ region of interest in the visual field, and the sum of the number of discrete robot arm joint movements in x, y, and z planes. However, the dependent variable analyzed in this study is the gaze data (gaze duration and fixation count).

## 4 Results

### 4.1 Learning Effect in the Use of Robot Controller

We investigated how frequently participants consulted the robot control instruction sheet as they repeated the task using gaze duration and the fixation count. Reduction in the frequency with which participants consulted the robot control instruction sheet as the task is repeated would imply that participants learned how to control the robot better with each task repetition.

**FIGURE 4:**
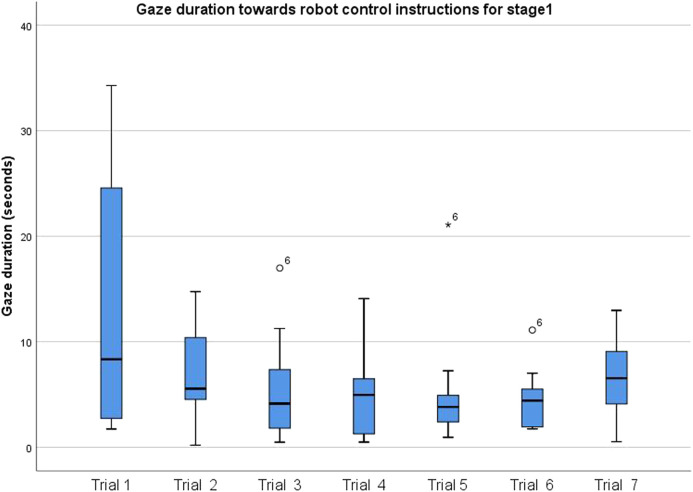
Gaze duration toward instructions as a function of attempt for stage 1.

To investigate whether the gaze duration for which participants looked at the instruction sheet changed with increasing familiarity, an ANOVA with a repeated measure of Attempt (attempt one to attempt seven) was conducted on the eye gaze duration toward the instruction sheet in stage one of the task. There was a trend with main effect of the Attempt order (F (2.08, 16.61) = 2.54, *p* = 0.107, *η* ^2^ = 0.241; Figure 4). The descriptive statistics indicate that indeed participants consulted the instructions less with each attempt they make, compared to the first attempt. In the follow-up analysis, we included participants’ gaming experience as a covariate. Gaming/robot control experience was considered to examine whether participants with prior experience would interact with the system different from those without prior gaming/robot control experience. The result showed a significant main effect of the Attempt order (F (6,30) = 3.63, *p* = 0.008, *η* ^2^ = 0.420), yet for the interaction Attempt order, gaming experience was not significant (F (6,30) = 1.29, *p* = 0.290, *η* ^2^ = 0.260). Equivalent analysis on scenarios (S1 to S7) did not show a significant main effect of feedback scenario without covariate and with covariate of gaming experience (F (6, 42) ≤ 0.90, *p* ≥0 .506, *η* ^2^ = 0.114). The results indicate that although gaze duration toward the instructions did not depend on feedback modalities, participants got more familiar and spent less time looking at instructions with task repetition. This difference was only at a trend level. This reached a significant result while considering participants’ gaming experience.

### 4.2 Gaze Duration and the Fixation Count Trend for Different Stages of the Task

**FIGURE 5:**
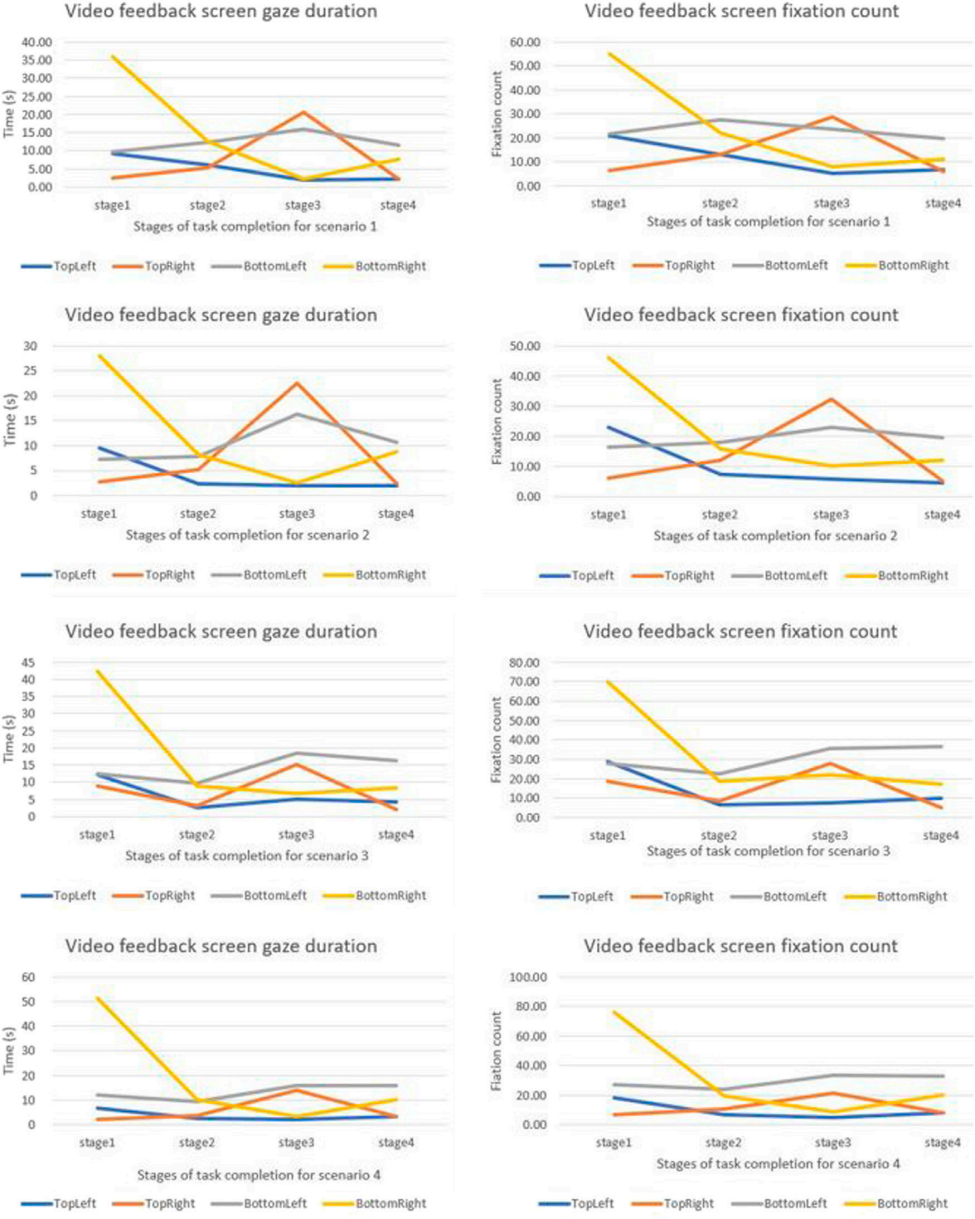
Graphical comparison of camera view changes for different stages of the task based on scenarios.

**FIGURE 6:**
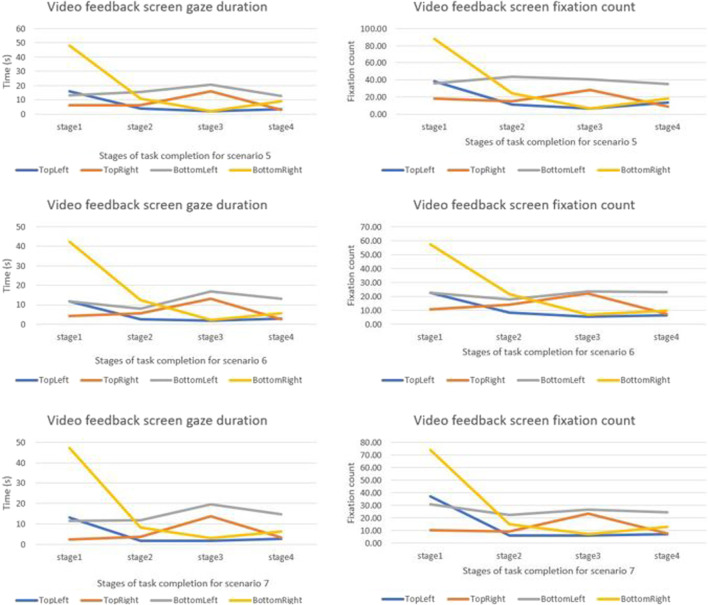
Graphical comparison of camera view changes for different stages of the task based on scenarios.

We compared how the gaze duration and the fixation count for different camera quadrants changed for different stages of the task. Gaze duration and the fixation count were highest in stage one and were observed to change for different stages of the task (Figure 5, 6). Neither the gaze duration nor the fixation count for any of the feedback quadrants yielded zero, implying that for all scenarios all the camera views were consulted notwithstanding the duration of consultation. For all the scenarios examined, gaze duration and the fixation count for the bottom-right quadrant were highest in the first task stage. The top-right quadrant for all scenarios had the highest gaze duration and the fixation count in the stage three of the task.

### 4.3 Effect of Feedback Modalities on Gaze View and Time

Next, we decided to investigate the camera quadrants that participants consulted the most, using the scenario with only video feedback (S1) as the control scenario. The repeated measures ANOVA was significant (F (1, 10) = 21.91, *p* = 0.001, *η* ^2^ = 0.687). Participants spent more time gazing at the bottom-right quadrant of the screen significantly more than the other three quadrants. Furthermore, gaze duration between other quadrants did not significantly differ (Table 3).

**TABLE 3:** Gaze duration mean, standard deviation (SD), and post hoc comparison between 4 angles significance level IN S1

	Mean	SD	N	Top left	Top right	Bottom left
Top left	9.32	8.79	11	-	-	-
Top right	2.54	5.38	11	0.249	-	-
Bottom left	9.72	6.87	11	≥0.999	0.212	-
Bottom right	35.93	16.19	11	0.007	0.001	0.002

To investigate the most consulted camera quadrant depending on feedback modality, a 4 (Camera Quadrant: top-left; top-right; bottom-left; bottom-right) x 7 (Scenarios: S1–S7) ANOVA with a covariate of gaming experience was conducted on the eye tracker gaze duration for each scenario. The results showed a significant main effect of the Camera Quadrant F (3,21) = 19.48, *p* ≤ 0.001, *η* ^2^ = 0.736, and main effect of Scenario (F (6, 42) = 2.41, *p* = 0.043, *η* ^2^ = 0.256). The interaction, Scenario x Camera quadrant, and interactions with the covariate of gaming experience did not yield a significant result F (6, 42) ≤ 1.85, *p* ≥ 0.113, *η* ^2^ = 0.209. Further investigation of main effect of camera quadrant showed that participants overall had a significantly longer gaze duration toward bottom-right quadrant compared to other quadrants (Table 4). This suggests that participants’ preference toward this quadrant was not significantly influenced by feedback scenarios.

**TABLE 4:** Gaze duration mean, standard deviation (SD), and post hoc comparison between 4 camera views significance level across all conditions (S1–S7).

	Mean	SD	N	Top left	Top right	Bottom left
Top left	10.87	8.53	9	-	-	-
Top right	3.33	2.92	9	0.185	-	-
Bottom left	11.87	9.49	9	≥0.999	0.0.173	-
Bottom right	41.61	18.95	9	0.028	0.004	0.010

Although the analysis yields the main effect of scenario to be significant, post hoc comparisons did not reveal participants having significantly longer gaze duration in any scenario (*p* ≥ 0.419). As the analysis shows that only the bottom-right quadrant was the most viewed, we further explored to see if different scenarios affect the viewing of this quadrant compared to the video-only scenario (S1). We compared the bottom-right quadrant of video-only scenario (S1) with other scenarios (S2–S7). The paired *t*-test between Scenario 1 (S1) and Scenario 4 (S4) was significant (t (10) = 2.74, *p* = 0.021), indicating that participants had significantly longer gaze duration toward the bottom-right quadrant in Scenario 4 (S4). Other comparisons were not significant (t (10) ≤ 1.62, *p* ≥ 0.136). When the relationship between gaze duration and participants’ performance on the task was examined for the first stage of the task, Pearson correlation coefficient established a significant positive correlation between gaze duration and the sum of individual robot joint movements (robot steps) (r = 0.715, *p* = 0.013).

Furthermore, the gaze duration at the bottom-right quadrant in Scenario 4 was the longest when compared to other scenarios. Descriptively, in this scenario, participants’ gaze duration in other quadrants was very low. Having longer gaze duration does not necessarily imply a corresponding higher fixation count, but the fixation count confirms the results of the gaze duration for this study.

### 4.4 Gaze Distractions by Feedback Modalities

Due to the importance of video feedback in teleoperation, as found in the results, we also sought to see the effect of each feedback scenario on participants’ gaze during the teleoperation task. We were interested in investigating whether the participants were distracted by the different feedback scenarios and the extent to which they were distracted. Distraction would be manifested as teleoperators looking away from areas of interest that they focused on when there wasn’t any other feedback (other than the video feeds). The areas of interest are defined as the screen for video feedback, the robot controller, and robot control instructions. The distraction time was calculated by subtracting the sum of gaze time on areas of interest from the total time of interest duration. The total time of interest duration is the time spent on the task. A repeated measures ANOVA with independent measure of seven scenarios and dependent measure of gaze duration outside areas of interest with covariant of gaming experience showed the main effect of scenario to be significant F (6,48) = 2.46, *p* = 0.037, *η* ^2^ = 0.235. The interaction Scenario x Gaming experience was not significant F (6, 48) = 1.95, *p* = 0.092, *η* ^2^ = 0.196.

Post hoc investigation with the Bonferroni adjustment for multiple comparison did not indicate any significant differences between scenarios (*p* ≥0 .999). The equivalent analysis on the attempt order showed that the main effect of attempt was significant (F (6,48) = 2.86, *p* = 0.018, *η* ^2^ = 263), as well as the interaction between attempt and gaming experience (F (6,48) = 3.11, *p* = 0.012, *η* ^2^ = 0.280). Further investigation of the main effect of attempt with Bonferroni adjustment for multiple comparisons did not indicate any significant difference between Attempts (*p* ≥0 .222). The result suggests that participants gaze outside the areas of interest was both dependent on feedback modalities (scenarios) and dependent on which attempt it was. However, post hoc results did not indicate significant difference between attempts and scenarios.

## 5 Discussion

As physically assistive robotic systems are still lacking in ability for safe fully autonomous operation, telerobotics can be employed to offer remote assistance. However, a relatively unexplored area of research, which this study addresses, is the understanding of how teleoperators use/relate with different feedback modalities and the teleoperation setup. Our experimental setup was designed for participants to carry out a teleoperated task of picking up a jar filled with sunflower seeds and emptying its content into another container. The task was repeated seven times for different feedback scenarios. In this study, we present the analysis of the gaze data recorded during the task to understand how teleoperators interacted with the system. Results on other findings during the same study have been published by Bolarinwa et al. (2019).

Results show that participants consulted the instruction sheet (on how to control the robot) less with each repetition of the task. However, the reduced consultation of the instruction sheet was not significantly affected by the different feedback scenarios or prior gaming experience of participants. This confirms the first hypothesis (H1) regarding a learning effect of robot control in teleoperated task repetition. Participants learn to control the teleoperated robot arm better with each repetition of the task. Even though proficiency improves with repetition, we were able to confirm that it can be monitored using gaze data and that feedback scenarios do not influence the learning process. The learning effect was not influenced by the feedback scenarios employed. The confirmation of hypothesis one (H1) is, however, relative to the specific joystick used in the study. Further studies can be carried out to confirm this hypothesis using other devices and technologies. The learning effect is also limited to how the robot is controlled without having any influence on how the task is carried out.

**FIGURE 7:**
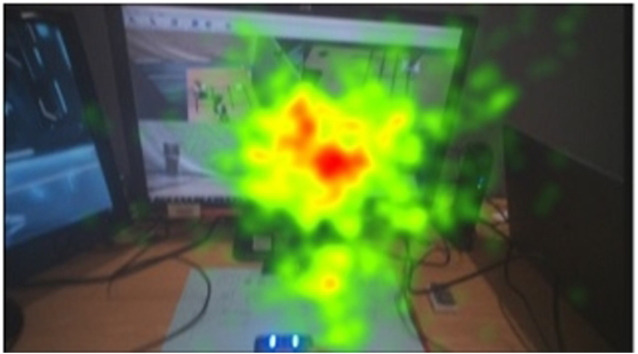
Heat map of average gaze data for stage 1 of the task.

Since the task carried out can be divided into stages, we examined gaze duration and the fixation count toward the different video feedback quadrants for the different stages of the task. Overall gaze duration and fixation count were observed to be highest in stage one of the task, relative to other stages. However, the least consulted video feedback quadrant cannot be concluded as the least important. Results show that all the video feedback quadrants were consulted but with varying gaze duration and the fixation count. Figure 7 shows the heat map of overall gaze duration for different camera quadrants. More studies may be carried out to examine the effect of removing the least consulted quadrants on the success of such teleoperated tasks. During stage three of the task, highest gaze duration and the fixation count were recorded toward the top right video feedback quadrant. Gaze duration and the fixation count for the bottom-right quadrant were highest in the first stage of the task. This suggests that participants prefer certain video feedback quadrants during specific stages of the task and this knowledge may be used to improve the design of video feedback for teleoperation. Video quadrant consultation can be influenced by the stage of the task. The sum of robot joint movements and objective evaluation of the different stages of the task show that more work was done in stages 1 and 3 of the task in order to complete those stages relative to stages 2 and 4. This was also confirmed with significantly higher gaze duration and the fixation count for stages 1 and 3 of the task.

The feedback scenarios employed did not influence the video feedback quadrant consulted, but for all scenarios, longer gaze duration and the fixation count were noticed for the bottom-right quadrant as participants carried out the task. Scenario 4 (S4) had the highest gaze duration when the effect of feedback was investigated for the bottom-right quadrant (Table 5). We compared the longer gaze duration and the fixation count results of Scenario 4 (S4) with results published by Bolarinwa et al. (2019). Bolarinwa et al. (2019) reported that the most significant improvement in gripper orientation was noticed for S4, and when the system usability scale was compared for all scenarios, S4 also had the joint highest score recorded. The gripper orientation is a measure of how accurately participants were able to orientate the gripper before grasping a jar for different feedback scenarios while the system usability scale is a subjective assessment of usability of the system for different feedback scenarios. Examining the number of robot joint steps needed to complete stage 1 across all scenarios and the ease of use according to Bolarinwa et al. (2019), S4 was reported to be the easiest scenario for participants.

**TABLE 5:** Gaze duration means (SD) across four camera views as a function of scenario for stage 1.

	Top left		Top right		Bottom left		Bottom right	
	Mean	SD	Mean	SD	Mean	SD	Mean	SD
S1	9.32	8.79	2.54	5.38	9.72	6.87	35.93	16.19
S2	9.64	12.62	1.95	4.50	7.41	5.71	26.78	9.16
S3	12.93	11.31	7.96	11.18	12.34	10.97	42.16	39.90
S4	6.85	7.04	2.14	3.80	11.69	10.75	49.39	26.63
S5	14.03	10.49	5.71	7.85	12.87	16.42	40.42	23.32
S6	12.61	12.36	4.80	4.35	12.33	15.51	39.62	30.54
S7	13.41	15.96	2.41	1.85	11.48	11.70	48.51	28.37

**FIGURE 8:**
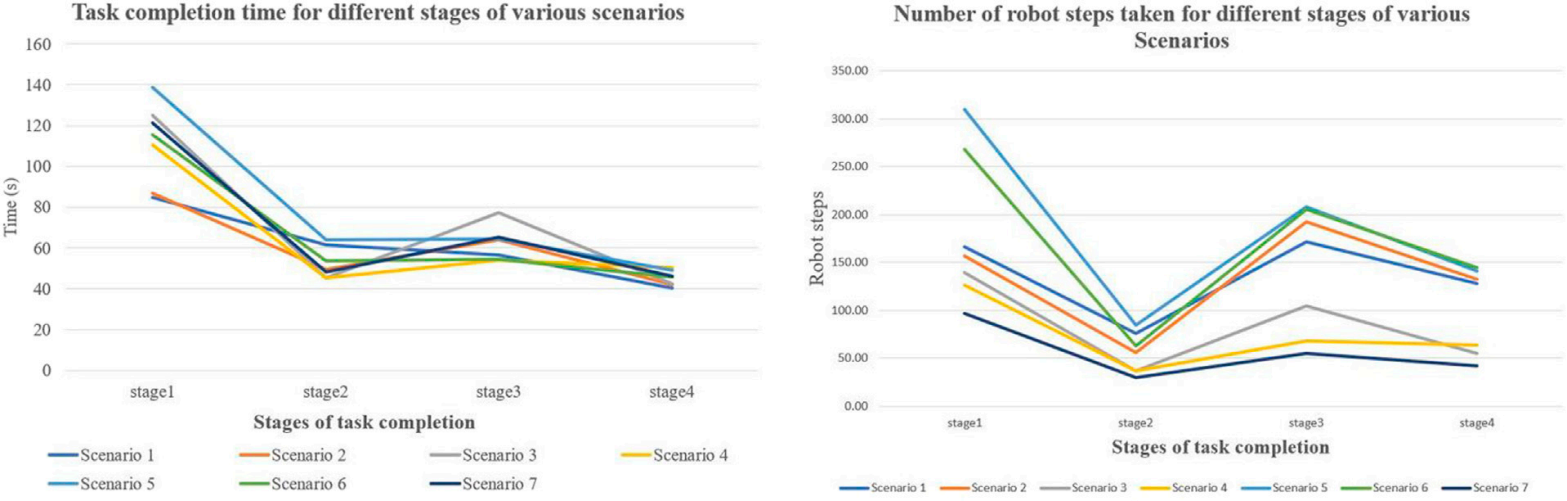
Task completion time and robot steps for all stages of the task.

The task completion time and the number of robot joint steps reflect a similar pattern to the gaze duration and the fixation count (Figure 8). The highest average task completion time and robot joint steps were recorded for stage 1, followed by stage 3. Subjectively examining the different stages of the task relative to the camera quadrants, we can infer that the bottom-right camera quadrant displays a better view of stage 1 of the task and that the top-right quadrant projects a better view of stage 3 of the task. This is confirmed by the fact that the bottom-right quadrant also had the highest gaze duration and the fixation count.

Results also show that gaze metrics can be used to infer the levels of distractions each feedback modality can introduce, confirming the third hypothesis (H3). In the study, there was no significant difference between the distractions introduced by the feedback scenarios.

With reference to the second hypothesis (H2), analysis of gaze duration and fixation count yields a significant main effect of scenario (combination of feedback). With no scenario having significantly longer gaze duration and the fixation count than other scenario, it is difficult to measure the impact of feedback scenarios on participants’ performance using gaze duration alone. However, since other parameters (e.g., distraction introduced by feedback scenarios and camera quadrants) that affect teleoperator performance can be measured using gaze duration and fixation count, it is difficult to ignore H2 but more studies may be carried out in this context. H2 is inconclusive in this study.

We can therefore infer the workload of different stages of the task by combining results for task completion time, gaze duration and fixation count, and robot joint steps. Stage 1 appears to be the stage with most workload. Stages 1 and 3 were found to possess more workload than stages 2 and 4 based on the recorded parameters. This type of analysis, which leads to gaining a better understanding of these relationships, can help with planning the optimal system setup for the teleoperator as it also gives an indication of the levels of cognitive workload for the various stages and aspects of the task. This information can further help to determine what support should be provided and at which point in the task execution it should be provided to ensure ease of teleoperator performance.

Whilst the results of the gaze data for this study did not indicate whether specific feedback modalities affect the participants’ gaze time on different camera views, recording gaze could be a useful method to determine a teleoperator’s preference of camera views in relation to the teleoperation task and its level of difficulty. This information could be very useful in taking an adaptive approach to the information being provided, for instance by enhancing the specific information, such as the magnification of a camera view, as the teleoperator proceeds with the task. The analysis of gaze data can also be used to confirm the effectiveness of each feedback modality. To further determine the significance of these findings, carrying out experiments with more participants will be needed. However, our experiments help to lay the foundations for the applicability of this approach.

## 6 Conclusion and Outlook

We examined the effect of feedback modalities on participants’ gaze. We also examined how gaze data can help us understand how teleoperators interact with the system, further helping to improve the system. We found that gaze data can be used to understand the areas of increased workload of various tasks and how it can be used to improve and optimize the setup and feedback modalities to suit the teleoperator. A key lesson learned is that, for assistive tasks, particularly when developing shared autonomy systems, analysis of gaze data of both the teleoperator and service user is likely to be important.

The analysis of gaze data could also be used to confirm the effectiveness of each feedback modality. Feedback on situational awareness could help to improve the accuracy with which a task can be carried out. However, processing feedback may also increase the task completion time. Since video feedback is provided across all feedback scenarios, any additional feedback (e.g., peripheral vision, haptic feedback, and verbal collaboration) increases the amount and type of information teleoperators have to process. It could therefore be inferred that additional feedback increases the cognitive load and may cause the increase in task completion time; however, more experiments will be required to establish this empirically. Also, even though repetition improves proficiency, with gaze metrics, we were able to confirm that feedback modalities did not influence the proficiency with which participants controlled the robot as they repeated the task. Understanding the distraction caused by the use of feedback may be important in scenarios where teleoperators need to focus on instantaneous changes at the remote end. In our experiments, gaze metrics confirmed that the use of additional feedback did not distract participants.

There is still a lot of anxiety expressed regarding the use of robots from both older adults and carers. In this study, we were, therefore, particularly interested in how the verbal collaboration could enhance the social elements of the interaction and perhaps reduce anxiety. As such, we explored the impact of verbal collaboration, as well as the efficacy of the feedback modalities for participants who might not have had prior experience of operating a robot arm. We observed how people without prior experience of robot control improved when verbal feedback was available. Based on the initial findings of this study, we recommend that this merits further exploration.

Further experiment with more participants, including older-adults as service-users to provide verbal feedback, and carers in the role of teleoperators, will help to improve the reliability of the findings. This will also enable us to determine the real-world applicability and utilization of this approach. While this study has helped to further understand not only the optimal setup for the teleoperator in regards to the feedback modalities, further research into the social interactions between service-users and teleoperators and how these might be affected by the cognitive workload on the teleoperator is required.

## Data Availability

The original contributions presented in the study are included in the article/Supplementary Material; further inquiries can be directed to the corresponding author.

## References

[B1] Begum M. Huq R. Wang R. Mihailidis A. (2015). Collaboration of an Assistive Robot and Older Adults With Dementia. Gerontechnology. 13, 405–419. 10.4017/gt.2015.13.4.005.00

[B2] Begum M. Wang R. Huq R. Mihailidis A. (2013). “Performance of Daily Activities by Older Adults with Dementia: The Role of an Assistive Robot,” in 2013 IEEE 13th International Conference on Rehabilitation Robotics (ICORR) (IEEE). 2013, 1–8. 10.1109/ICORR.2013.6650405 24187224

[B3] Bolarinwa J. Eimontaite I. Dogramadzi S. Mitchell T. Caleb-Solly P. (2019). “The Use of Different Feedback Modalities and Verbal Collaboration in Tele-Robotic Assistance,” in 2019 IEEE International Symposium on Robotic and Sensors Environments (ROSE), 1–8. 10.1109/ROSE.2019.8790412

[B4] [Dataset] Brooke J. (2004). Japan Seeks Robotic Help in Caring for the Aged. Caring. 23, 56. Available at: https://www.nytimes.com/2004/03/05/world/machida-journal-japan-seeks-robotic-help-in-caring-for-the-aged.html . 10.7275/e4r6-dj05 15341304

[B5] Carreto C. Gêgo D. Figueiredo L. (2018). An Eye-Gaze Tracking System for Teleoperation of a Mobile Robot. J. Inf. Syst. Eng. Management. 3, 16. 10.20897/jisem.201816

[B6] Chen J. Ji Z. Niu H. Setchi R. Yang C. (2019). An Auto-Correction Teleoperation Method for a Mobile Manipulator Using Gaze Tracking and Hand Motion Detection. Towards Autonomous Robotic Systems,Lecture Notes Computer Sci. 11650, 422–433. 10.1007/978-3-030-25332-5_36

[B7] Gidlöf K. Wallin A. Dewhurst R. Holmqvist K. (2013). Using Eye Tracking to Trace a Cognitive Process: Gaze Behaviour during Decision Making in a Natural Environment. J. Eye Movement Res. 6, 1. 10.16910/jemr.6.1.3

[B8] Góngora Alonso S. Hamrioui S. de la Torre Díez I. Motta Cruz E. López-Coronado M. Franco M. (2019). Social Robots for People With Aging and Dementia: a Systematic Review of Literature. Telemed. e-Health. 25, 533–540. 10.1089/tmj.2018.0051 30136901

[B9] [Dataset] Hernandez U. M. Boorman L. Prescott T. (2017). Multisensory Wearable Interface for Immersion and Telepresence in Robotics. IEEE Sensors J. 17, 2534. 10.1109/jsen.2017.2669038

[B10] Huang C.-M. Andrist S. Sauppé A. Mutlu B. (2015). Using Gaze Patterns to Predict Task Intent in Collaboration. Front. Psychol. 6, 1049. 10.3389/fpsyg.2015.01049 26257694 PMC4513212

[B11] Jacob R. J. K. (1990). “What You Look at Is what You Get: Eye Movement-Based Interaction Techniques,” in Proceedings of the SIGCHI Conference on Human Factors in Computing Systems (New York, NY, USA: Association for Computing Machinery), 11–18. 10.1145/97243.97246

[B12] Jones R. M. Wray R. E. Zaientz J. Bachelor B. Newton C. (2015). Using Cognitive Workload Analysis to Predict and Mitigate Workload for Training Simulation. Proced. Manufacturing. 3, 5777–5784. 10.1016/j.promfg.2015.07.825

[B13] [Dataset] Kinova (2019). KINOVA JACO Assistive Robotic Arm. Available at: https://www.kinovarobotics.com/en/assistive-technologies/column-a1/kinova-assistive-robotic-arm

[B14] Kosch T. Hassib M. Woźniak P. W. Buschek D. Alt F. (2018). “Your Eyes Tell,” in Proceedings of the 2018 CHI Conference on Human Factors in Computing Systems (New York, NY, USA: Association for Computing Machinery), 1–13. 10.1145/3173574.3174010

[B15] Langer A. Feingold-Polak R. Mueller O. Kellmeyer P. Levy-Tzedek S. (2019). Trust in Socially Assistive Robots: Considerations for Use in Rehabilitation. Neurosci. Biobehavioral Rev. 104, 231–239. 10.1016/j.neubiorev.2019.07.014 31348963

[B16] Latif H. O. Sherkat N. Lotfi A. (2009). “Teleoperation Through Eye Gaze (TeleGaze): A Multimodal Approach,” in 2009 IEEE International Conference on Robotics and Biomimetics (ROBIO), 711–716. 10.1109/ROBIO.2009.5420585

[B17] Loth S. De Ruiter J. P. (2016). Editorial:Understanding Social Signals: How Do We Recognize the Intentions of Others?. Front. Psychol. 7, 281. 10.3389/fpsyg.2016.00281 26941706 PMC4763189

[B18] Luz R. Corujeira J. Silva J. L. Ventura R. (2018). “Traction Awareness Through Haptic Feedback for the Teleoperation of UGVs,” in 2018 27th IEEE International Symposium on Robot and Human Interactive Communication (New York, United States: RO-MAN), 313–319. 10.1109/ROMAN.2018.8525740

[B19] Lv H. Yang G. Zhou H. Huang X. Yang H. Pang Z. (2020). Teleoperation of Collaborative Robot for Remote Dementia Care in Home Environments. IEEE J. Transl. Eng. Health Med. 8, 1–10. 10.1109/jtehm.2020.3002384 PMC7326153 32617197

[B20] Mizukoshi Y. Sato R. Eto T. Kamezaki M. Matsuzaka A. Yang L. (2020). “A Low Cognitive Load and Reduced Motion Sickness Inducing Zoom Method Based on Typical Gaze Movement for Master-Slave Teleoperation Systems With HMD,” in 2020 IEEE/SICE International Symposium on System Integration (SII), 28–33. 10.1109/SII46433.2020.9026260

[B21] Pamungkas D. Ward K. (2015). “Immersive Teleoperation of a Robot Arm Using Electro-Tactile Feedback,” in 2015 6th International Conference on Automation, Robotics and Applications (ICARA), 300–305. 10.1109/ICARA.2015.7081164

[B22] Triantafyllidis E. Mcgreavy C. Gu J. Li Z. (2020). Study of Multimodal Interfaces and the Improvements on Teleoperation. IEEE Access. 8, 78213–78227. 10.1109/ACCESS.2020.2990080

[B23] Tsitsimpelis I. Taylor C. J. Lennox B. Joyce M. J. (2019). A Review of Ground-Based Robotic Systems for the Characterization of Nuclear Environments. Prog. Nucl. Energ. 111, 109–124. 10.1016/j.pnucene.2018.10.023

[B24] Vitense H. S. Jacko J. A. Emery V. K. (2002). “Multimodal Feedback,” in Proceedings of the Fifth International ACM Conference on Assistive Technologies (New York, NY, USA: Association for Computing Machinery), 49–56. 10.1145/638249.638260

[B25] Wang Z. Reed I. Fey A. M. (2018). “Toward Intuitive Teleoperation in Surgery: Human-Centric Evaluation of Teleoperation Algorithms for Robotic Needle Steering,” in 2018 IEEE International Conference on Robotics and Automation (ICRA), 5799–5806. 10.1109/ICRA.2018.8460729

[B26] Wickens C. D. (2002). Multiple Resources and Performance Prediction. Theor. Issues Ergon. Sci. 3, 159–177. 10.1080/14639220210123806

[B27] William S. (2018). Medical Definition of Peripheral Vision. Available at: https://electronics.howstuffworks.com/everyday-tech/haptic-technology.htm#pt2

[B28] Winter J. (2013). Using the Student’s “t”-Test With Extremely Small Sample Sizes. Pract. Assess. Res. Eval. 18, 10. 10.7275/e4r6-dj05

